# AEGIS—AcutE Geriatric Intervention Study: pilot study of frontline acute geriatric assessment to improve quality of care in emergency department

**DOI:** 10.1093/ageing/afae171

**Published:** 2024-08-08

**Authors:** Kaisa J Karjalainen, Hannele Tuori, Marika Salminen, Juha Peltonen, Sirpa Rantanen, Paula Viikari, Matti Viitanen, Maria S Nuotio, Laura Viikari

**Affiliations:** Department of Geriatric Medicine, Faculty of Medicine/Clinical Medicine, University of Turku and Turku University Hospital, The wellbeing services county of Southwest Finland, Turku, Finland; Department of Geriatric Medicine, Faculty of Medicine/Clinical Medicine, University of Turku and Turku University Hospital, The wellbeing services county of Southwest Finland, Turku, Finland; Tyks Acute/Turku University Hospital, The wellbeing services county of Southwest Finland, Turku, Finland; Department of Geriatric Medicine, Faculty of Medicine/Clinical Medicine, University of Turku and Turku University Hospital, The wellbeing services county of Southwest Finland, Turku, Finland; Department of General Practice, Faculty of Medicine/Clinical Medicine, University of Turku and The wellbeing services county of SouthwestFinland; Tyks Acute/Turku University Hospital, The wellbeing services county of Southwest Finland, Turku, Finland; Tyks Acute/Turku University Hospital, The wellbeing services county of Southwest Finland, Turku, Finland; Department of Geriatric Medicine, Faculty of Medicine/Clinical Medicine, University of Turku and Turku University Hospital, The wellbeing services county of Southwest Finland, Turku, Finland; Department of Geriatric Medicine, Faculty of Medicine/Clinical Medicine, University of Turku and Turku University Hospital, The wellbeing services county of Southwest Finland, Turku, Finland; Division of Clinical Geriatrics, Center for Alzheimer Research, Department of Neurobiology, Care Sciences and Society, Karolinska Institutet and Karolinska University Hospital Huddinge, Stockholm, Sweden; Department of Geriatric Medicine, Faculty of Medicine/Clinical Medicine, University of Turku and Turku University Hospital, The wellbeing services county of Southwest Finland, Turku, Finland; Department of Geriatric Medicine, Faculty of Medicine/Clinical Medicine, University of Turku and Turku University Hospital, The wellbeing services county of Southwest Finland, Turku, Finland; Tyks Acute/Turku University Hospital, The wellbeing services county of Southwest Finland, Turku, Finland

**Keywords:** geriatric emergency medicine, emergency department, frailty, targeted geriatric assessment, geriatrician-led team, older people

## Abstract

**Introduction:**

Due to the increasing number of older patients in emergency departments (EDs) with frailty, cognitive impairment and multimorbidity, there is a need for geriatric expertise in EDs.

**Methods:**

This retrospective study is of older patients visiting Turku University Hospital ED between 2 January and 31 December 2022. Patients aged 75 years of older were screened for frailty using Triage Risk Screening Tool (TRST) and Clinical Frailty Scale (CFS). Nonacute, frail patients (CFS ≥4) suitable for Targeted Geriatric Assessment (TGA) (*n* = 1096) were scanned for the risk of delirium, cognitive impairment, change in functional status, falls, malnutrition and depression. A comprehensive patient record was made with recommendations for future care.

**Results:**

TRST was completed in 70% of the ED visits, and two-thirds of those were considered high-risk. Among the patients assessed by the geriatric team (TGA), nonspecific complaint (38%) and falls (35%) were the main reasons for ED admission. Cognitive impairment was present in over 60% and orthostatic hypotension in 40% of the patients. The 72-hour revisit rate for TGA-patients was 2.3%. For the real-life control group, the 72-hour revisit rate was 4.6% (*P* = .001). Thirty-day revisit rates were 10% and 16%, respectively (*P* < .001). The need for rehabilitation, cognitive evaluation and intensifying home care were the main recommendations for future care.

**Conclusions:**

TGA approach provides structured and accurate information on older patients’ background. This may lead to more precise diagnostics, a thorough consideration of hospital intake and a secure discharge from the ED. Ensuring continuity of care may help to reduce readmissions to EDs.

## Key points

- The majority of the patients aged 75 years or older admitted to the emergency department were acutely ill; only in a minority of cases were the reasons for the visit solely social.
- The Targeted Geriatric Assessment provides more structured and accurate information on the functional capacity and background of older patients, thus leading to more accurate diagnostics, a thorough consideration of hospital intake and a secure discharge from the emergency department.
- The need for rehabilitation, cognitive evaluation and intensifying home care were the main recommendations for future care.
- Ensuring continuity of care may help to reduce readmissions to emergency departments.

## Background

With an ageing population and increasing longevity, we are facing rising number of older patients in all nonpaediatric medical specialties and also in emergency departments (EDs) [1]. A recent Finnish study reported that 15% of ED visits are made by patients that are at least 80 years old [2]. According to a recent collaboration study, the prevalence of frailty in European EDs has been estimated to be 40% [3].

As multimorbidity and frailty increase with age, the last few years of life will become the most expensive [4]. A symptom-oriented and organ-specific approach is ill-suited to older patients with nonspecific complaints; instead, they benefit from a targeted geriatric assessment and holistic care. Acute exacerbations of chronic conditions in frail multimorbid patients struggling to manage at home will lead to an increased use of ED services and hospital admissions [5].

The first guidelines for geriatric emergency care were published in 2014 by emergency physicians, nurses and geriatricians [6]. In 2020, three models of emergency geriatric care were presented in a review that stated that the existing guidelines are poorly implemented [7]. Updated guidelines for geriatric emergency care were published in 2022 [8]. Although these guidelines emphasise the need for geriatric competence, the evidence on the effectiveness of geriatric interventions in ED settings is still inconclusive. However, there is a tendency towards positive outcomes [9, 10].

Frail older patients often present in the ED with nonspecific complaints or falls. Identifying orthostatic hypotension, underlying cognition deficit, risk of delirium or potentially harmful medications may reduce revisit rates to EDs and hospitalisation. At best, it may also shorten the length of stay in hospital [5, 8].

A variety of different risk predictor tools have been created to identify frail older patients in busy ED settings [11]. The Triage Risk Screening Tool (TRST) is a validated six-item questionnaire predicting risk of ED revisits, hospital admissions and nursing home admissions of older patients [12]. The cut-off score of 2 points is considered high risk.

A multidisciplinary acute geriatric team was piloted in Turku University Hospital ED as a part of a nationwide health care reform in autumn 2021. Turku University Hospital is a tertiary hospital serving 470 000 inhabitants (year 2021). The ED provides both specialist acute care and primary care acute services 24/7 for Turku and 27 other cities and municipalities in southwestern Finland. The objectives of the pilot were, primarily, to improve the quality of the ED care for older patients by indicating the risks and special features, such as delirium, frailty and medication-related issues. Secondly, the objective was to avoid unnecessary hospital admissions often leading to functional decline, and thirdly, to ensure seamless follow-up care, blood test controls, evaluation of the effects of possible medication changes and identifying the need for further geriatric evaluation and rehabilitation.

In this paper, we describe the design of the Acute Geriatric Intervention Study (AEGIS) and present the preliminary results. The AEGIS study aims to describe:

- the proportion of people aged 75 years or older in ED who were at risk of frailty [Clinical Frailty Scale (CFS) 4 or over] and who are likely to benefit from targeted geriatric assessment (TGA),
- the basic characteristics of these patients,
- the proportion of the patients who were medically too emergent to be assessed and
- the frequency of the nonmedical (i.e. social) reasons underlying the primary reason for the ED visit (failure demand, underlying need for care, unmet needs).

## Material and methods

**Figure 1:**
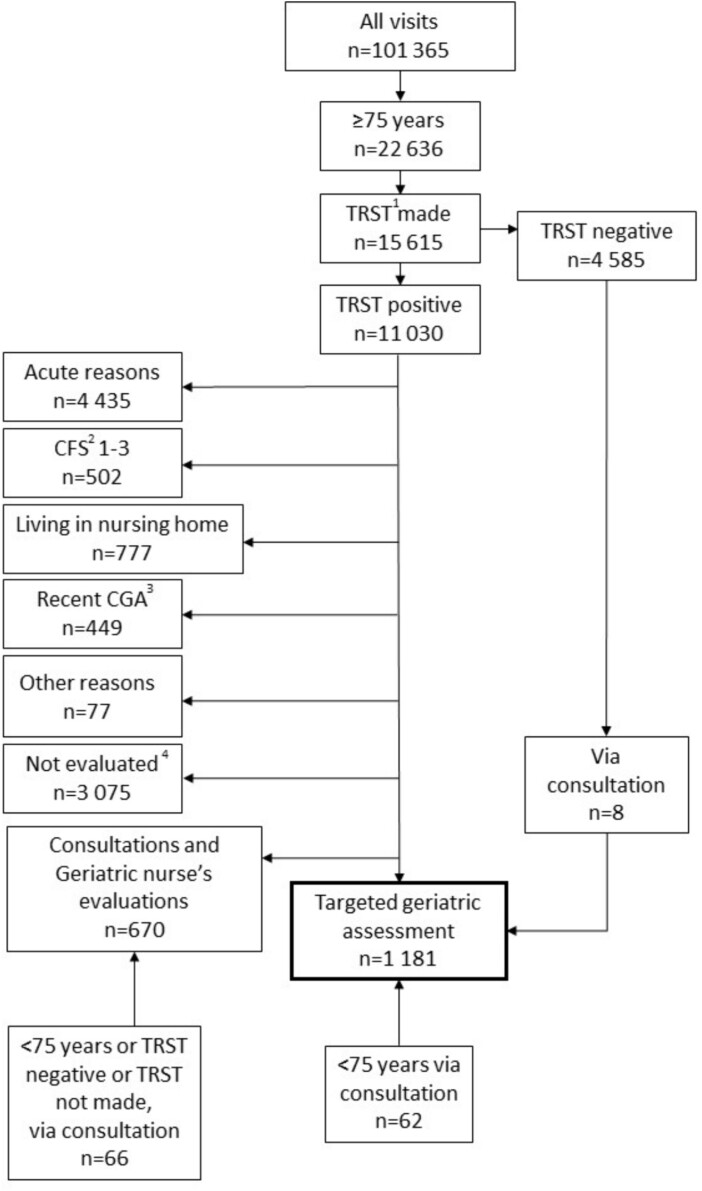
The flow chart of the study. ^1^TRST = Triage Risk Screening Tool. ^2^CFS = Clinical Frailty Scale. ^3^CGA = Comprehensive Geriatric Assessment. ^4^TRST-positive patients, who were not assessed by the geriatric team were retrospectively evaluated to rule out patients who would not have been eligible for geriatric evaluation. The remaining patients form a real-life control group (*n* = 2642) for TGA patients. For further information on patients not assessed, please see Appendix 1, supplementary data available in *Age and Ageing* online.

This is a retrospective study of patients aged 75 years or older visiting Turku University Hospital ED during 2 January to 31 December 2022. Data were collected from the patient records via an automated computer database search. The flow chart of the study is shown in Fig. 1.

### AEGIS—AcutE Geriatric Intervention Study

The acute geriatric team worked from Monday to Friday in two shifts between 8 a.m. and 8 p.m., with two geriatricians and three to four acute geriatric nurses. During the weekends, an acute geriatric nurse worked from 10 a.m. to 6 p.m. without a geriatrician.

Patients aged 75 years and older were screened by triage nurse, paramedics or an emergency care nurse by using the TRST. During the implementation and education period of the TRST tool, a majority of the TRST was made by geriatric team nurses and doctors, either by studying the patient records or by interviewing the patient.

The patients with imminent life-threatening or acute medical situation were not eligible for TGA. The acuteness of the patients’ situation was evaluated by using the National Early Warning Score (NEWS2) [13]. Three points on one parameter or a total score of five points or over was interpreted as an acute condition. Patients with an acute chief complaint such as myocardial infarct, stroke, hip fracture, seizure, acute surgical problem or septic infection were also excluded.

**Figure 2:**
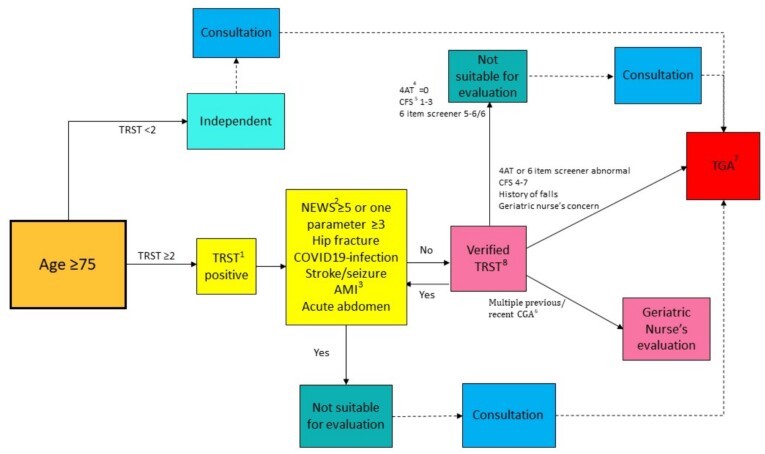
The protocol of the patient selection. ^1^TRST = Triage Risk Screening Tool. ^2^NEWS = National Early Warning Score. ^3^AMI = acute myocardial infarct. ^4^4AT = 4 A’s Test. ^5^CFS = Rockwood Clinical Frailty Scale. ^6^CGA = Comprehensive Geriatric Assessment. ^7^TGA = Targeted Geriatric assessment. ^8^Verified TRST = nonacute, TRST-positive patients were assessed by the acute geriatric nurse to estimate the functional status and CFS of the patient.

The non-acute, TRST-positive patients were further interviewed by the acute geriatric nurse to estimate the functional status and CFS of the patient (verified TRST) [14]. The modified Activities of Daily Living and Instrumental Activities of Daily Living (ADL-IADL) questionnaire [15] was used. In addition, delirium (4 A’s Test, 4AT) [16] and cognitive status tests (Six-item Screener) [17] were made. The patients with cognitive impairment, risk of delirium or a CFS of 4–7 were assessed by the geriatric team (nurse and geriatrician), and a full TGA was made. Patients under 75 years with a need for a geriatric consultation could also be referred to the geriatric team for evaluation. Patients with a recent history of a comprehensive geriatric assessment (CGA) or TGA and patients living in nursing homes were excluded from TGA, unless a referral for consultation was made by ED staff. The protocol of the patient selection is shown in Fig. 2.

Not all eligible patients could be assessed, due to the limited resources and patients being admitted to ED outside of the operating time of the team. The International Classification of Primary Care (ICPC2) codes of these TRST-positive patients were retrospectively checked to determine their reason for admission. Patients presenting an acute chief complaint were excluded, as well as the patients who were deceased or not from the Southwest Finland hospital district area. The remaining patients (*n* = 2642) were used as a real-life control group to compare the results of the TGA to the standard ED care. The flow chart of the control group is shown in Appendix 1, and reasons preventing the TGA are listed in Appendix 2; data are available in *Age and Ageing* online.

The TGA contained questions concerning risk of falls, incontinence, malnutrition, depression, alcohol abuse and smoking. In addition, a brief questionnaire concerning the quality of life (EuroHis-8) was also made [18]. The medication was thoroughly ascertained by the acute geriatric nurse to obtain a thorough insight of any recent changes made to the medication and to evaluate if there were any alterations in the functional ability that could be related to the medication. Of uttermost importance was to acquire reliable information about the patient’s prior functional ability. The answer to the question ‘What has changed and in what time?’ was essential to align the intensity of the diagnostic tests, to assess the medication and delineate the treatment and make the decision to admit or discharge the patient. A special questionnaire was developed to ensure a structured assessment of the patient (Appendix 3); supplementary data are available in *Age and Ageing* online.

At the beginning of the pilot, the ED doctor responsible for the care of the patient was informed of the observed problems. However, during the pilot, it became increasingly apparent that the frail and old benefit from a more holistic approach of geriatrician-led TGA. Therefore, the team shifted towards the team geriatrician also taking charge of the acute medical care of the patient. A comprehensive patient record was made of the assessment and the findings. The recommendations for future care were communicated directly to the social and primary care facilities of the patient’s home municipality via phone or secured e-mail to ensure continuity of care after the ED visit.

### Ethics

The study was conducted according to the guidelines of the Declaration of Helsinki. The authorisation for the study was obtained from the Hospital District of Southwest Finland Ethics Committee. Since the study was register-based, signed consent was not required.

## Results

Overall, 101 365 visits were made in the ED during 2 January 2022 to 31 December 2022. Of these, 22 636 (22%) were made by patients 75 years or older. TRST was completed in 15 615 visits (69%), of which 11 030 visits (71%) were defined as high-risk (TRST-positive). TGA was not considered suitable in 6240 TRST-positive cases. Acute reasons, such as acute myocardial infarct, stroke, or hip fracture, were the most common reasons preventing TGA (71%). Verified TRST showed 5% of the TRST-positive patients as being non-frail (CFS 1–3) (Figs 1 and 2.)

### Results of targeted geriatric assessment

The TGA was performed at 1181 visits (1096 individual patients). Fifty-seven patients were evaluated twice and 14 patients three times during the year. In addition, an acute geriatric nurse’s evaluation or a shorter consultation was performed at 670 visits. Sixty-two patients under 75 years old were referred to geriatric consultation by the ED staff. The characteristics of TGA patients are presented in Table 1 and the characteristics and future recommendations and actions of TGA visits in Table 2.

**Table 1:** Patient characteristics according to the Targeted Geriatric Assessment (*n* = 1096)

Sociodemographics	Mean (SD), range/median (IQR), range	*n* (%)
Age (years), mean (SD), range	84.9 (6.7), 61–105	
Female		733 (67)
Living conditions		
Alone		744 (68)
With a spouse		289 (26)
With somebody else		38 (4)
Nursing home		24 (2)
Data not available		1 (0)
Home care		
None		620 (57)
Less than once a day		102 (9)
Once a day		98 (9)
Twice a day		130 (12)
At least three times a day		141 (13)
Family caregiver		90 (8)
Data not available		5 (1)
Functional capacity		
Clinical Frailty Scale, mean (SD), range	5.6 (1.3), 1–9	
Charlson Comorbidity Index		
0		107 (10)
1		224 (20)
2		224 (20)
3		193 (18)
≥4		348 (32)
Number of chronic diseases, median (IQR), range	5 (4–7), 0–14	
Number of medications, median (IQR), range	8 (6–11), 0–26	
Use of mobility aid		
Yes		798 (73)
No		262 (24)
Data not available		36 (3)
History of falls (previous 6 months)		
Yes		702 (64)
No		336 (31)
Data not available		58 (5)
Cognition deficit		
None		430 (39)
Mild cognitive impairment or cognition deficit without diagnosis		296 (27)
Diagnosed dementia		370 (34)

**Table 2:** Characteristics of the emergency department visits according to the Targeted Geriatric Assessment and Geriatric interventions (*n* = 1181).

Characteristics of the ED visits	*n* (%)
Way of accessing ED	
Ambulance	746 (63)
Referral	103 (9)
Triage	332 (28)
Chief complaint	
Specific	326 (28)
Nonspecific	447 (38)
Falls	408 (35)
Duration of symptoms	
Acute (1–3 days)	847 (72)
Subacute	280 (24)
Chronic (>3 months)	54 (5)
Postdischarge plan	
Home	474 (40)
Hospital admission	642 (54)
Other facilities	64 (5)
Not known	2 (0)
4AT	
0	508 (43)
1–3	447 (38)
4–12	189 (16)
Not assessed	37 (3)
Six-item Screener	
Normal	374 (32)
Abnormal	744 (63)
Not assessed	63 (5)
Orthostatic hypotension	346 (29)
Not assessed	308 (26)
Inproper medication	371 (31)
Nonmedical reason for admission	11 (1)
Inadequate home care	439 (37)
Inadequate home care leading to ED admission	224 (19)
Geriatric interventions	
Medication alterations	625 (53)
Starting/levelling up home care	459 (39)
Referral to comprehensive geriatric assessment	159 (14)
Referral to rehabilitation	233 (20)
Referral to memory clinic	133 (11)
Follow-up in primary care	84 (7)
Referral to another specialty	85 (7)

The TGA patients were frail, multimorbid and multimedicated. Thirty-eight percent presented nonspecific complaints, such as feeling unwell or exhausted, generalised weakness, dizziness, gait disturbances and confusion, and 35% had fallen or had recurrent falls as a reason for admission to ED. The majority (72%) had acute onset of symptoms, and only 5% presented with chronic symptoms. Fifty-four percent of the patients were admitted to hospital care.

One-third (34%) of the patients had a dementia diagnosis, and, in addition, 27% had symptoms of cognitive decline, but had not yet been properly diagnosed. Six-item Screener score was abnormal in 63% and delirium risk according to 4AT was presented in 16% of the assessments. Orthostatic hypotension was tested in three out of four TGA cases, and 43% of the tests made were positive. Potentially harmful medication or improper use of medication was present in 31% of the cases. Non-medical reason for ED admission was found only in 11 cases (1%). However, indications of inadequate home care could be identified in 37% of the cases, and, in 19% of the cases, this was a direct cause of the medical problem presented in ED (Table 2.)

### Geriatric interventions

Alterations in medications were made in slightly over half (53%) of the cases; 20% of the patients were referred to physiotherapy or rehabilitation, and for 39% of the patients, new or additional home care was arranged after the ED visit. Eleven percent of the patients were referred to a memory clinic, and, additionally, 14% were referred to further CGA in a geriatric ward or outpatient clinic (Table 2).

### Revisits and length of geriatric assessment

The revisit rates were analysed for the patients aged 75 years or older who were discharged home from the index visit. Of the TGA visits, 2.3% (*n* = 26/1119) had a revisit within 72 hours; the 30-day revisit rate was 9.5% (*n* = 106/1119). In comparison, among the control group, the 72-hour revisit rate was 4.6% (*n* = 121/2642) (*P* = .001) and the 30-day revisit rate 15.7% (415/2642) (*P* < .001). (Table 3.)

**Table 3:** Revisit rates in different patient groups

Revisit rate	I Patients aged 75 years and over with TGA^a^ (*n* = 1119) *n* (%)	II All patients aged 75 years and over, not assessed by the geriatric team (*n* = 20 890) *n* (%)	III TRST^b^-positive, nonacute patients aged 75 years and over, not assessed by the geriatric team (*n* = 2642) *n* (%)	*P*-value^c^ I vs. II	*P*-value^c^ I vs. III
72 hours	26 (2.3)	642 (3.1)	121 (4.6)	.154	.001
30 days	106 (9.5)	2155 (10.3)	415 (15.7)	.365	<.001

^a^TGA , Targeted Geriatric Assessment

^b^TRST, Triage Risk Screening Tool

^c^Chi-square test

It was not possible to extract the exact length of geriatric assessment from the digital patient information system.

## Discussion and conclusions

This multidisciplinary geriatric frontline assessment pilot, started in autumn 2021, has since proven to follow the most recent published guidelines [8]. According to the TGA, older patients admitted to ED were frail, multimorbid and multimedicated. One-third had previously been diagnosed with dementia and, in addition, almost one-third had cognitive impairment, but were lacking a proper diagnosis. Therefore, the background history collected from home care and relatives is of uttermost importance, both when considering the examinations needed and making the postdischarge plan [19].

The TGA is a structured approach, which is beneficial especially with nonspecific or clinically atypical symptoms; often, the ‘rule-out worst-case scenario’ is suboptimal [1] and may lead to parsimony. It is important not to accept a single explanation, for example, a urinary tract infection, as being the sole cause of a complex situation [19]. In our study, falls and nonspecific complaints were the most common reasons for ED admissions of the TGA patients; 35% presented in the ED with a fall or recurrent falls, and 64% had a history of falls in the previous 6 months. Falls are associated with higher morbidity and mortality, poorer overall functioning and earlier admission to long-term care. Preventing future falls with referral to rehabilitation, a thorough evaluation of medications and active screening of orthostatic hypotension is considered beneficial [20]. In our TGA patients, orthostatic hypotension was a frequent finding. A Finnish study has shown adverse drug events being associated with 20% of ED admissions in older patients [21]. Recent guidelines for falls prevention call for opportunistic case finding and personalised multidomain interventions for high-risk patients to reduce further risk for falls. Frail patients presenting in ED with falls are therefore an especially potential group for the TGA approach [22].

Identifying vulnerable ED patients who would benefit from a geriatric evaluation would improve the quality of care of complex multimorbid older patients, and to a certain extent, improve ED flow. A ‘Silver-line’ from triage to geriatric ED unit in urgent, nonacute situations would allow a more patient-centred and secure way to treat these patients [5]. TRST is one of the standard instruments for screening older patients benefiting from geriatric assessment. In our study, however, it seemed to be inaccurate in recognising independent patients with CFS 1–3. Two-thirds of the patients aged 75 years or over were TRST-positive but verified TRST showed 5% being independent. Therefore, additional verification of the patient’s vulnerability using the CFS or other frailty tools is needed when screening patients benefiting from geriatric evaluation. Using only CFS as a screening tool was not considered suitable due to its modest screening concordance [23]. Other triage screening tools may be more suitable, and this should be further evaluated [11].

The data collected are real-life data, which acts both as a strength and a weakness. All older adults visiting the ED were potential targets of TGA, if they met the criteria. On the other hand, the limited personnel resource made the team vulnerable, especially during rush hours, and the team could only assess one-third of the potentially eligible patients. Although TRST was implemented in the digital patient information system, there was some reluctance to complete the questionnaire, probably due to ED overcrowding and a lack of understanding of the benefits of the geriatric assessment. Consequently, almost one-third of the older patients did not have TRST screening. Unfortunately, limited background information in the register data on the control patients prevented an actual case–control setting, a method which would strengthen the findings of this study.

During the period under review, there was a chronic lack of hospital beds in the Hospital District of Southwest Finland, which led to extended lengths of stays in the ED. Although the data from the digital patient information system were not precise on the length of the geriatric assessment, at least in this context, TGA did not excessively lengthen the ED stay. On the contrary, after a frontline geriatric assessment, it is possible to safely discharge the nonacute frail patients with tailored treatment plans, thus saving them from overcrowded EDs, prolonged ED stays [24] and unnecessary hospital admissions, all of which are known to be detrimental for this group of patients [25]. The patients evaluated by the acute geriatric team, although representing the most frail, multimorbid and multimedicated older individuals visiting the ED, had a significantly lower revisit rates both 72 hours and 30 days after the index visit, when compared to nonacute patients aged 75 years or older with a positive result in the TRST screening. However, due to our retrospective approach, it is not possible to reliably determine the effect of TGA on the revisits and the results have to be therefore interpreted with caution.

A multiprofessional, holistic approach with skilled acute geriatric nurses working closely together with geriatricians ensures the best possible outcome and the postdischarge treatment plan in situations where other options besides admission to hospital ward may be considered. The cost-effectiveness of this approach must be further evaluated [26].

Patients being admitted to the ED due to nonmedical (i.e. social) reasons was the original premise for this pilot. In our study, 99% of the TGA visits were found to have a medical problem leading to ED admission. In addition, an underlying lack of care was found in 37% of the TGA visits, and in 19% of the visits, it was considered to be the direct cause of the medical reason leading to ED admission. Insufficient home care, when unnoticed, may lead to failure demand, revisits to the ED, possibly unnecessary hospital admissions, and at worst, the loss of functionality and even the need for future long-term care [27].

The TGA approach will provide more structured and accurate information on the functional capacity of older patients as well as more reliable background data on acute symptoms and the reason for the ED visit. This may lead to more accurate diagnostics, more consideration before hospital intake and a secure discharge from the ED. Ensuring continuity of care may also help to reduce ED readmissions. Geriatricians and acute geriatric nurses working on the front line are thus protecting the old and frail patients from diagnostics and treatment that are either over- or underestimated and making sure that resilient, fit and independent older individuals are treated medically effectively despite their chronological age.

## Supplementary Material

aa-24-0332-File002_afae171
