# Supplementary material for: AEGIS—AcutE Geriatric Intervention Study: pilot study of frontline acute geriatric assessment to improve quality of care in emergency department
Source: Age Ageing. 2024 Aug 8;53(8):afae171. doi: 10.1093/ageing/afae171 (PMC11306315; doi:10.1093/ageing/afae171)

**AEGIS - AcutE Geriatric Intervention Study: Pilot study of frontline acute geriatric assessment to improve quality of care in Emergency Department**

Supplementary Data

Contents list

Appendix 1. Flow chart of control group; the TRST-positive, non-acute patients aged 75 and over, not assessed by acute geriatric team.

Appendix 2. Reasons preventing Targeted Geriatric Assessment.

Appendix 3. The TGA form.

Appendix 4. The multi-professional targeted geriatric assessment.

Appendix 1. Flow chart of the control group; the TRST-positive, non-acute patients aged 75 and over, not assessed by acute geriatric team.

^1^ TRST = Triage Risk Screening Tool

^2^ TGA = Targeted Geriatric Assessment

^3^ICPC2 = International Classification of Primary Care, 2^nd^ edition


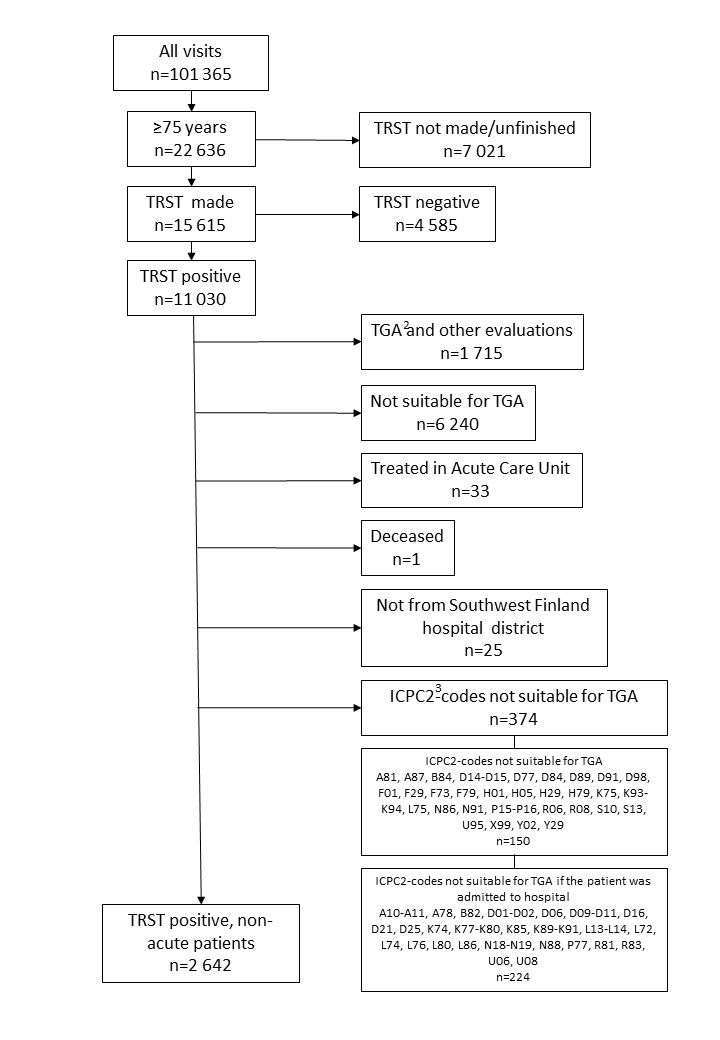


|  |  |  |  |
| --- | --- | --- | --- |

Appendix 2. Reasons preventing Targeted Geriatric Assessment.

^1^ NEWS = National Early Warning Score

^2^ CFS = Rockwood Clinical Frailty Scale

^3^ CGA = Comprehensive Geriatric Assessment

|  | n (%) |
| --- | --- |
| **Acute reasons** | **4 435 (71%)** |
| Stroke / seizure | 267 |
| Acute infection / COVID-19 | 1 473 |
| Acute cardiopulmonary disease | 329 |
| Acute abdomen | 211 |
| Hip fracture | 190 |
| NEWS^1^ ≥5 or one parameter ≥3 | 716 |
| Other surgical acute problem | 637 |
| Acute psychosis | 30 |
| Under the influence of alcohol | 24 |
| Referral for acute imaging | 181 |
| Other acute situations | 377 |
|  |  |
| **Other reasons** | **1 805 (29%)** |
| CFS^2^ 1-3 | 502 |
| Previous / Recent CGA^3^ | 449 |
| Living in long term care | 777 |
| Other reasons | 77 |

Appendix 3. The TGA form.

Appendix 4. The multi-professional targeted geriatric assessment.

^1^ ADL-IADL = Activities of Daily Living – Instrumental Activities of Daily Living

^2^ 4AT = 4 A’s Test

^3^ QOL = Quality of Life

^4^ VAS = Visual Analogue Scale

^5^ CFS = Rockwood Clinical Frailty Scale


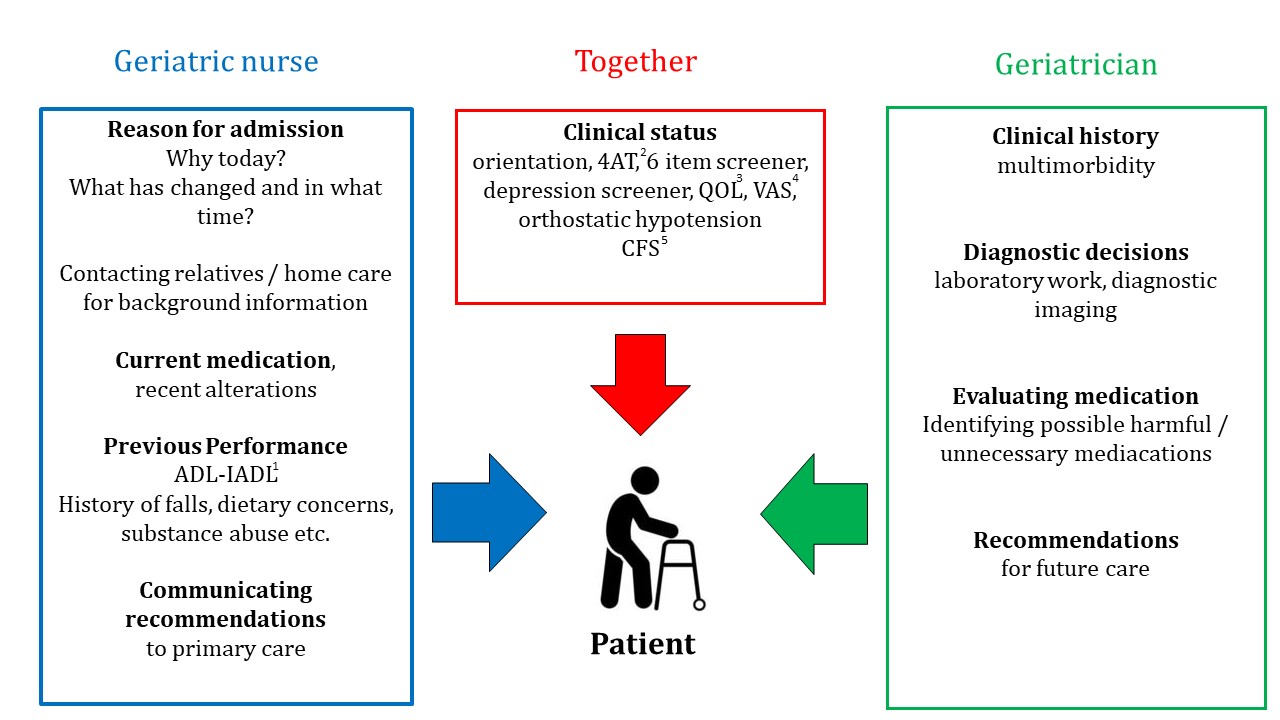

Supplement: aa-24-0332-File002_afae171 [file aa-24-0332-file002_afae171.docx]
